# THE VAGINAL MICROBIOTA BEFORE AND AFTER HYPEROSMOLAR LUBRICANT USE IN PRE-, PERI-, AND POSTMENOPAUSAL WOMEN

**DOI:** 10.1093/geroni/igae098.2404

**Published:** 2024-12-31

**Authors:** Sarah Brown, Xin He, Elizabeth Johnston, Katrina Mark, Jacques Ravel, Rebecca Brotman

**Affiliations:** University of Maryland School of Medicine, Baltimore, Maryland, United States; University of Maryland, College Park, Maryland, United States; University of Maryland Baltimore, Baltimore, Maryland, United States; University of Maryland School of Medicine, Baltimore, Maryland, United States; University of Maryland School of Medicine, Baltimore, Maryland, United States; University of Maryland School of Medicine, Baltimore, Maryland, United States

## Abstract

Vaginal lubricants are commonly used in clinical practice and to alleviate vulvovaginal symptoms in menopause. However, most products are hyperosmolar and may disrupt beneficial vaginal bacteria like Lactobacillus spp. We sought to investigate the impact of a single exposure to lubricant among patients referred for transvaginal ultrasound (TVUS) in which lubricant was used during the exam. We recruited 104 participants who self-collected mid-vaginal swabs daily for one week between baseline and TVUS, and immediately before and 6-12 hours after TVUS. Participants attended a visit 2-5 days after TVUS, continued to sample twice weekly, and attended a final clinical visit in week 10. Microbiota composition was characterized by 16S rRNA gene amplicon sequencing and assigned to community state types (CSTs; low-Lactobacillus CST-IV vs Lactobacillus-dominated CSTs I/II/III/V). Yue-Clayton theta indices defined vaginal microbiota stability before and after TVUS. Generalized linear mixed models evaluated the within-participant relative change in the odds of having a CST-IV microbiota after TVUS versus before. Our findings suggest that vaginal microbiota stability decreased after TVUS. Despite no overall change in the odds of a CST-IV microbiota after TVUS among all participants, certain subgroups showed increased risk. Specifically, peri/postmenopausal women (N=19, aOR:3.22, 95% CI:1.16-8.98) and those with a history of bacterial vaginosis (N=58, aOR:1.73, 95% CI:1.10-2.72) were more prone to decreases in Lactobacillus-dominant microbiota after TVUS. In contrast, premenopausal women and those with no bacterial vaginosis history appeared more resilient against perturbations. Reformulation may be necessary to reduce lubricant toxicity, particularly for peri/postmenopausal women who frequently use these products.

